# Identification of an eleven-miRNA signature to predict the prognosis of endometrial cancer

**DOI:** 10.1080/21655979.2021.1952051

**Published:** 2021-08-01

**Authors:** Jing Lu, Jianqiang Liang, Mengting Xu, Zhipeng Wu, Wenjun Cheng, Jie Wu

**Affiliations:** a State Key Laboratory of Reproductive Medicine, Department of Obstetrics and Gynecology, The First Affiliated Hospital of Nanjing Medical University, Jiangsu Province Hospital, Jiangsu Women and Children Health Hospital, Nanjing, China; b Department of Gynecology, the First Affiliated Hospital of Nanjing Medical University, Nanjing 210029, Jiangsu province, China; c Department of Urology, the Affiliated Sir Run Run Hospital of Nanjing Medical University, Nanjing 221116, Jiangsu province, China

**Keywords:** Endometrial cancer, TCGA, miRNA, model, prognosis

## Abstract

Endometrial cancer (EC) is the most common gynecological malignancy. Recent studies have uncovered miRNA acted a striking role in predicting the prognosis of multiple tumors. Over 500 EC samples were selected from the Cancer Genome Atlas (TCGA) database. Univariate, LASSO and multivariate Cox regression analysis were employed to screen out the prognosis-involved miRNAs. Kaplan-Meier (K-M) and time-dependent receiver operation characteristic (ROC) curves were conducted to reveal survival analysis and assess the accuracy of the signature. The independence of the model was verified via univariate and multivariate Cox regression analysis. Besides, qRT-PCR was conducted to testified the expression of 11 miRNAs in 16 paired tissues. A total of 514 specimens were randomly divided into the training set and the testing set, then an 11 miRNAs-based signature were determined which divided the patients into high-risk group and low-risk group. The survival was markedly different and the ROC curve exhibited a precise prediction. Meanwhile, the univariate and multivariate Cox regression analysis verified the miRNAs-based model was an independent indicator of EC. Moreove, the prediction ability of this model with clinicopathological features was more efficient. Finally, functional enrichment analysis demonstrated these miRNAs were associated with the occurrence and progression of cancer. Additionally, hsa-mir-216b, hsa-mir-363, hsa-mir-940 and hsa-mir-1301 were highly expressed in EC tissues in contrast to normal tissues through qRT-PCR. Importantly, the eleven-miRNA signature was full of robust ability to predict the prognosis of EC.

## GRAPHICAL ABSTRACT

**Figure:**
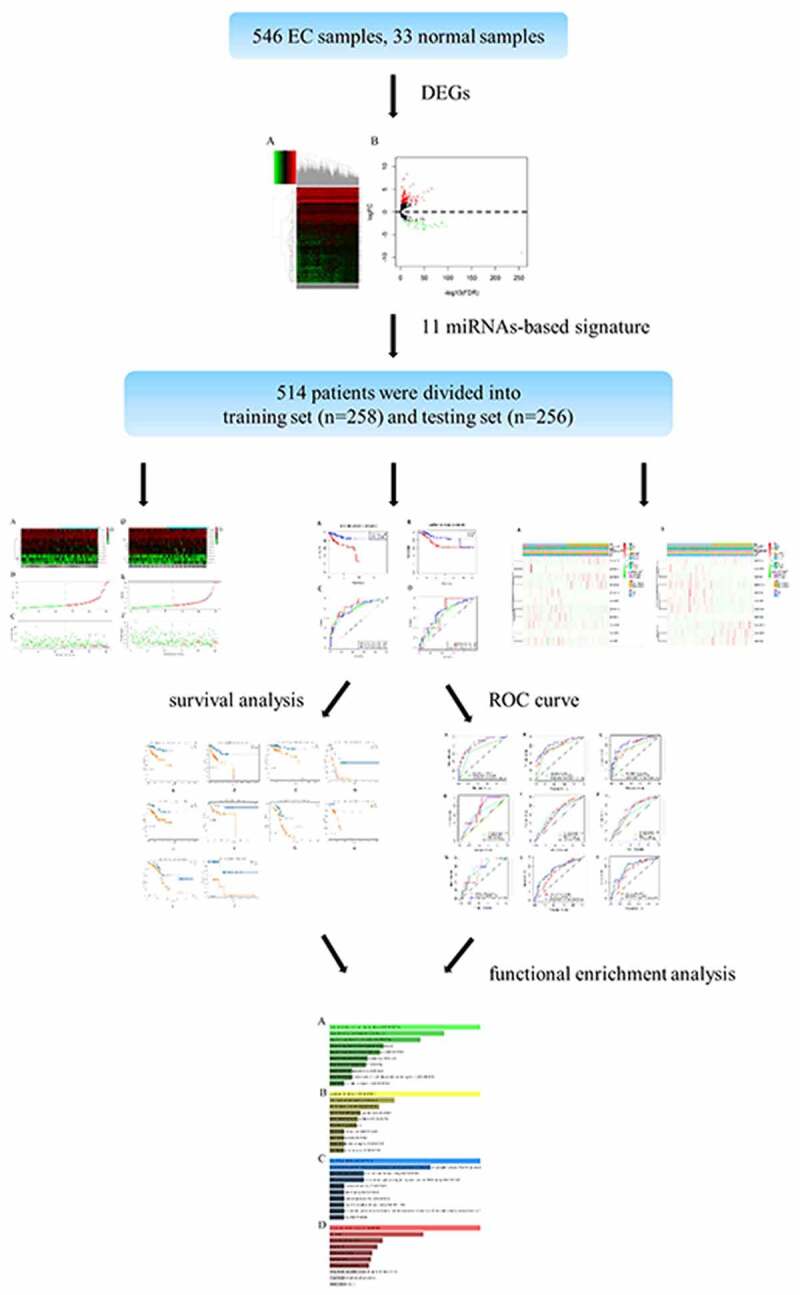

## 1. INTRODUCTION

Endometrial cancer (EC) is one of the most malignant tumors derived from epithelial tissue that seriously threatens women’s health, the morbidity of it among females ranked the fourth [1]. There are multiple factors that influenced morbidity and mortality, such as the histology (serous) and the grade (grade 3) impacted the 5-year survival rate, which was the lowest [2]. Besides, intentional weight loss was verified to contribute to an apparent low risk of type I endometrial cancer [3]. Although there was a prominent decline in postoperative mortality from 2000–2001 (0.70%) to 2008–2009 (0.48%) [4], and the adjuvant radiation treatment (ART) significantly improved disease-free survival (DFS), overall survival (OS) in patients with early-stage EC [5], patients with metastasis had a poor prognosis, only with 17% of 5-year survival [6]. In recent years, various biomarkers were discovered associated with the tumorigenesis [7], progression [8], prognosis [9–11] of EC, but still tough to apply. Therefore, it is pressing to construct a systemic model to estimate prognosis by integrating these biomarkers or some unknown.

MicroRNAs which can target mRNAs mostly through interaction with the 3′UTR [12] and regulate the expression of genes are short RNA molecules 19 to 25 nucleotides in size [13]. Moreover, the miRNA acts as an adaptor for miRISC to recognize or regulate mRNA and the product (miRNA-mRNA) was a key determinant of the regulatory mechanism [14]. Recent studies hinted miRNAs were involved in far-ranging diseases, such as cancer, diabetes, viral infections and others [15]. Among them, the cancer-related miRNAs had sprung up. For example, overexpression of miR24-2 accelerates the progression of liver cancer cells through Pim1 activation [16], while downregulated miR-28-5p in prostate cancer acts as a tumor suppressor by altering SREBF2, which is a vital mediator of miR-28-5p [17]. For endometrial cancer, plenty of relevant microRNAs and their mechanisms were disclosed. Some typical examples as following: highly-expressed miRNA-486-5p induced proliferation and migration of EC cells through targeting MARK1 [18]; high expression of miR-137 was considered as a tumor suppressor to inhibit the cell proliferation by targeting EZH2 and LSD1 [19]; Similarly, highly-expressed miR-449a attenuated cell growth and metastasis based on the regulatory axis of NDRG1/PTEN/AKT [20]. Despite these miRNAs were explored in EC, their reliability and stability were controversial. Hence, to establish a reliable prognosis-related signature was urgent to address so as to guide the subsequent treatment for the heterogeneous individual and promote survival after the operation or receiving comprehensive therapy.

In this study, we obtained miRNAs data and clinical information from TCGA database and construct a miRNA-based signature to explore its role in prediction of EC prognosis. Our hypothesis is that miRNA-based model has the robust ability to predict EC prognosis. The aim of our study is to establish a novel model that integrated biomarkers with clinicopathologic features to evaluate the prognosis of EC patients accurately. Our goal is to lay the theoretical basis for further pursuing a new therapeutic strategy to enhance the survival rate.

## 2. MATERIALS AND METHODS

### 2.1 Selection of patient datasets

In this study, a total of 579 samples, which contained 546 EC patients and 33 normal tissues, were obtained from The Cancer Genome Atlas (TCGA) database (https://TCGAData.nci.nih.gov/TCGA/) [21]. First, according to the integrity of the miRNA expression profile and clinicopathological features, 514 samples were ensured for subsequent analysis. Secondly, we acquired the clinical information including prognostic information, age, grade, stage and so on. Finally, 514 samples were randomly divided into training sets (n = 258) and testing sets (n = 256).

### 2.2 Prognosis-involved miRNAs screening

EdgeR package [22] in R language was executed to screen out all differentially expressed miRNAs (DEMs) between 546 EC patients and 33 adjacent normal samples. The volcano plot based on the inclusion criteria (|log2FC|>1 and FDR<0.05) was emerged, then cluster analysis was conducted to demonstrate the evident difference between two antagonistic sets intuitively. After that, Univariate, LASSO, and multivariate Cox regression analysis were applied to select the prognosis-involved miRNAs orderly in the training sets. Notably, based on the candidate miRNAs, which were acceptable to *P* < 0.05 in univariate Cox regression analysis, the OS-related miRNAs could be further screened out via LASSO, multivariate Cox regression analysis.

### 2.3 Establishment of a multi-miRNA-based prognostic model

After using the LASSO and multivariate Cox regression analysis, the most valuable miRNAs were ascertained. Based on these miRNAs, the prognostic risk stratification was revealed by calculating the risk score in the training sets. Risk score, where *Coef_i_* represented the coefficient of miRNA_i_, *Exp_i_* denoted the expression lever of miRNA_i_. The median score was served as optimal cutoff value to separate the cohort into high- and low-risk group, then we compared survival rate between the two groups through Kaplan-Meier(K-M) survival curves and verified the predictive efficiency via time-dependent receiver operation characteristic (ROC) curves. To validate the validity of the results, the multi-miRNA-based risk score was applied in the testing sets, and the K-M curves and ROC curves were also performed to investigate whether the results were steady.

### 2.4 Survival analysis and comparison of the miRNAs expression profile

Firstly, we performed the different distribution of clinicopathological features between the high-risk group and the low-risk group via the Chisq-test. Then we divided those samples into different subgroups according to clinicopathological variables. K-M curves were built to analyze the overall survival of the high-risk group and the low-risk group of every subgroup in the training cohort and testing cohort respectively. Subsequently, the t-test was utilized to manifest the significant difference in the expression profile of DEMs in different subgroups.

### 2.5 Verification of model’s dependence and assessment of its prediction accuracy

Univariate and multivariate Cox regression analyses were employed to verify the miRNA-based prognostic model was independent and estimate the hazard ratio of each risk factor in the training set and testing set. The area under the curve (AUC) of time-dependent ROC was calculated to compare the accuracy of the different factors or their combinations in predicting 1-, 3-, 5-year survival in the training cohort. Naturally, the AUC of time-dependent ROC was executed in the testing cohort to validate the verdict obtained from the training cohort likewise.

### 2.6 Functional enrichment analysis

We utilized the TargetScan, miRTarBase and miRDB to infer the target genes regulated by those miRNA. After that, the Enrichr database (http://amp.pharm.mssm.edu/Enrichr/) [23] was applied to elaborate GO and KEGG pathways closely implicated in those target genes. Through functional enrichment analysis, it implied the internal biological mechanisms of these target genes. Besides, the GO analysis contained three categories: biological process (BP), cellular component (CC) and molecular function (MF).

### 2.7 RNA extraction and quantitative reverse transcription PCR (qRT-PCR)

Trizol reagent (Invitrogen) was applied to extract total RNA from 16 paired tissues (16 endometrial cancer tissues and 16 normal tissues) which were obtained from the First Affiliated Hospital of Nanjing Medical University (Nanjing, China). PrimeScript^TM^ RT Reagent Kit (Takara Bio, Inc., Otsu, Japan) was used for reverse transcription (RT). SYBR PrimeScript RT PCR kit (Jijia, Suzhou, China) was employed for qRT-PCR. Additionally, U6 was seved as an internal reference. The primer sequences of 11 miRNAs were predented in Table 1. The comparative cycle threshold (2^−ΔΔCt^) were analyzed to acquired the final results via GraphPad Prism 7. Notably, our study was approved by the Ethics Committee of the First Affiliated Hospital of Nanjing Medical University.

**Table 1.** The primer sequences for 11 miRNAs

miRNA	primer sequence
hsa-mir-216b-F	ACACTCCAGCTGGGAAATCTCTGCAGGCAA
hsa-mir-216b-R	CTCAACTGGTGTCGTGGAGTCGGCAATTCAGTTGAGTCACATTT
hsa-mir-592-F	ACACTCCAGCTGGGTTGTGTCAATATGCGA
hsa-mir-592-R	CTCAACTGGTGTCGTGGAGTCGGCAATTCAGTTGAGACATCATC
hsa-mir-3170-F	ACACTCCAGCTGGGCTGGGGTTCTGAGACA
hsa-mir-3170-R	CTCAACTGGTGTCGTGGAGTCGGCAATTCAGTTGAGACTGTCTG
hsa-mir-215-F	ACACTCCAGCTGGGATGACCTATGAATTG
hsa-mir-215-R	CTCAACTGGTGTCGTGGAGTCGGCAATTCAGTTGAGGTCTGTCA
hsa-mir-940-F	ACACTCCAGCTGGGAAGGCAGGGCCCCCG
hsa-mir-940-R	CTCAACTGGTGTCGTGGAGTCGGCAATTCAGTTGAGGGGGAGCG
hsa-mir-3614-F	ACACTCCAGCTGGGCCACTTGGATCTGAAGG
hsa-mir-3614-R	CTCAACTGGTGTCGTGGAGTCGGCAATTCAGTTGAGGGGCAGCC
hsa-mir-1301-F	ACACTCCAGCTGGGCGCTCTAGGCACC
hsa-mir-1301-R	CTCAACTGGTGTCGTGGAGTCGGCAATTCAGTTGAGTGCTGCGG
hsa-mir-363-F	ACACTCCAGCTGGGCGGGTGGATCACGATG
hsa-mir-363-R	CTCAACTGGTGTCGTGGAGTCGGCAATTCAGTTGAGAAATTGCA
hsa-mir-4687-F	ACACTCCAGCTGGGCAGCCCTCCTCCCGCA
hsa-mir-4687-R	CTCAACTGGTGTCGTGGAGTCGGCAATTCAGTTGAGTTTGGGTG
hsa-mir-96-F	ACACTCCAGCTGGGTTTGGCACTAGCACATT
hsa-mir-96-R	CTCAACTGGTGTCGTGGAGTCGGCAATTCAGTTGAGAGCAAAAA
hsa-mir-7110-F	ACACTCCAGCTGGGTGGGGGTGTGGGGAG
hsa-mir-7110-R	CTCAACTGGTGTCGTGGAGTCGGCAATTCAGTTGAGCTCTCTCT

## 3. RESULTS

In this study, we construct an miRNA-based signature to explore its role in prediction of EC prognosis based on TCGA database. We hypothesized that miRNA-based model has the robust ability to predict EC prognosis. Our goal is to construct a miRNA-based signature to predict prognosis of EC and to lay the theoretical basis for further pursuing a new therapeutic strategy to enhance the survival rate. In the present study, we first obtained over 500 EC samples were selected from the Cancer Genome Atlas (TCGA) database. Then we screened out most significantly expressed miRNAs and constructed a signature. The predictive ability was estimated and validated. Besides, we combined miRNA-based model with clinicopathological features to assess the prediction ability of different combinations. Finally, the biological functions were predicted by GO and KEGG.

### 3.1 Differentially expressed miRNAs screening

**Figure 1.**
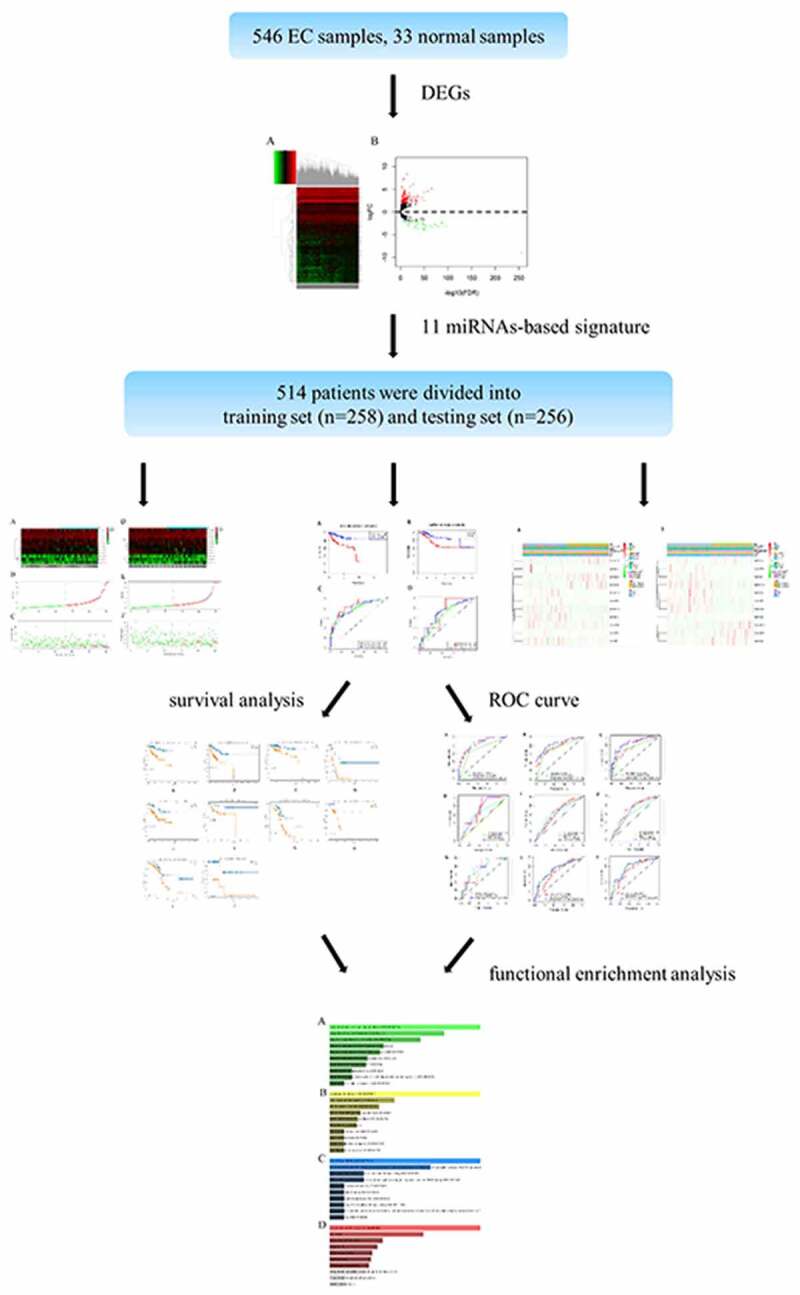
Identification of the differentially expressed miRNAs. A, Volcano plot specifies the inclusion criteria of DEMs: |log2FC|>1 and FDR<0.05. B, Heatmap depicts the expression of DEMs among all samples

A total of 579 samples in this study were composed of 546 endometrial cancer (EC) patients and 33 adjacent normal samples. It was apparent that a total of 196 significantly differentially expressed miRNAs comprised of 151 up-regulated miRNAs and 45 down-regulated miRNAs (inclusion criteria: |log2FC|>1 and FDR<0.05) between EC patients and normal samples were displayed in the volcano plot (Figure 1a). From the heatmap, we could perceive all DEMs and the clustering analysis among all specimens (Figure 1b).

### 3.2 Identification of prognosis-related miRNAs

In the training cohort, by performing univariate Cox regression analysis, a total of 19 miRNAs were served as the candidate miRNAs (*P* < 0.05). LASSO Cox regression analysis shrank the figure from 19 to 17 miRNAs. Ultimately, multivariate Cox regression analysis was performed to screen out the most prominent DEMs, which could be served as a prognostic model. Consequently, 11 miRNAs were confirmed (Table 2), including eight up-regulated miRNAs (hsa-mir-216b, hsa-mir-592, hsa-mir-215, hsa-mir-940, hsa-mir-1301, hsa-mir-363, hsa-mir-96, hsa-mir-7110) and 3 down-regulated miRNAs (hsa-mir-3170, hsa-mir-3614, hsa-mir-4687).

**Table 2.** The most striking DEMs identified via multivariate Cox regression analysis

Id	Coef	HR	HR.95 L	HR.95 H	P
hsa-mir-216b	0.035811618	1.036460577	1.016858357	1.056440675	0.000236884
hsa-mir-592	0.001139494	1.001140143	1.000490689	1.001790019	0.000578198
hsa-mir-3170	−0.027016017	0.973345651	0.946717071	1.000723221	0.056277372
hsa-mir-215	0.000708305	1.000708555	1.000385564	1.001031651	0.000017
hsa-mir-940	0.016577139	1.016715302	1.005229596	1.028332243	0.004239174
hsa-mir-3614	−0.014393591	0.985709501	0.971924728	0.999689784	0.045161922
hsa-mir-1301	0.00287944	1.00288359	0.999441393	1.006337642	0.100706384
hsa-mir-363	0.000206199	1.00020622	1.000035561	1.000376909	0.017864718
hsa-mir-4687	−0.196972793	0.821212976	0.650347133	2.000376909	0.097935417
hsa-mir-96	0.001700232	1.001701678	0.999940819	3.000376909	0.058220406
hsa-mir-7110	0.317116657	1.373162752	1.080406223	4.000376909	0.009538667

### 3.3 Establishment of EC prognostic model in the training set

We integrated miRNA expression profiles and clinical information so as to identify 514 eligible EC samples, then we divided these samples into the training set (n = 258) and the testing set (n = 256) randomly.

According to the formula: Risk score, the specific risk score for individuals were calculated as following: Risk score = (0.035811618 × expression level of hsa-mir-216b) + (0.001139494 × expression level of hsa-mir-592) + (−0.027016017 × expression level of hsa-mir-3170) + (0.000708305 × expression level of hsa-mir-215) + (0.016577139 × expression level of hsa-mir-940) + (−0.014393591 × expression level of hsa-mir-3614) + (0.00287944 × expression level of hsa-mir-1301) + (0.000206199 × expression level of hsa-mir-363) + (−0.196972793 × expression level of hsa-mir-4687) + (0.001700232 × expression level of hsa-mir-96) + (0.317116657 × expression level of hsa-mir-7110).

**Figure 2.**
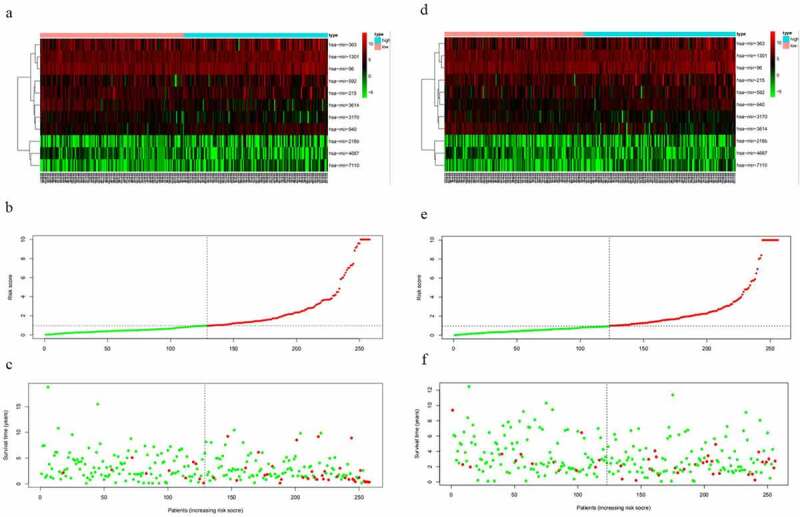
The variance of the expression level of DEMs, risk score, and survival status between high-risk set and low-risk set. A-C, The variance in the training set. D-F, The difference in the testing set

**Figure 3.**
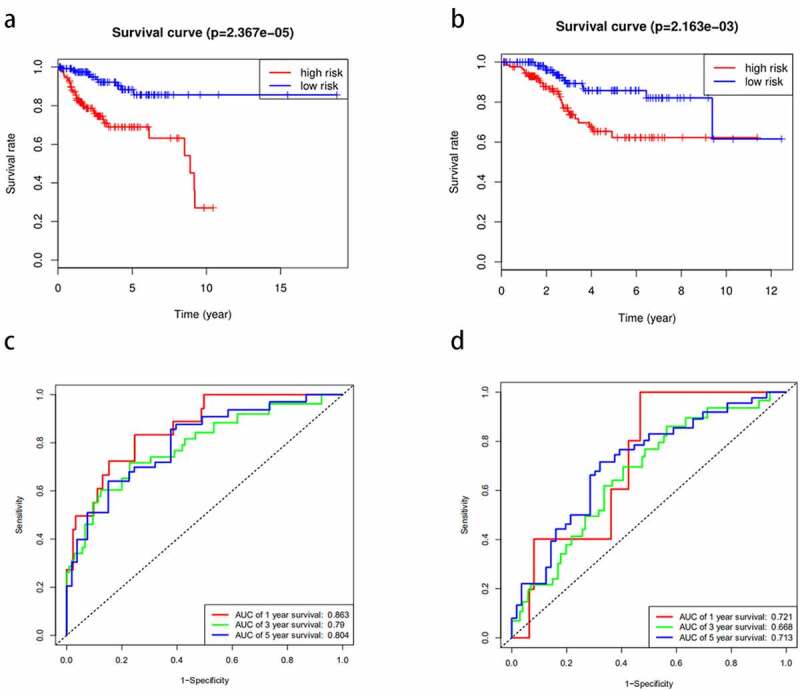
The prognostic model to predict the survival of the EC. Kaplan–Meier survival curve unveils the survival rate of the high-risk group and low-risk group in the training set (a) and testing set (b). ROC curves testifies the precision of the predictive ability in the training set (c) and testing set (d)

From the above, we acknowledged that coefficient < 0 mean a better prognosis, while coefficient > 0 indicated a poor prognosis. The median risk score among risk scores of all samples in the training cohort was served as the optimal cutoff value, which separated the patients into high- and low-risk group in the training cohort. The expression level of 11 miRNAs, risk score, and survival status were displayed in Figure 2a-2c. The survival rate of the high-risk group was conspicuously poorer than that of the low-risk group via the K-M analysis (*P* = 2.367e^−5^) (Figure 3a). ROC analysis was conducted to assess the accuracy of the prognostic ability (Figure 3c). The area under the ROC curve (AUC) was 0.863 at 1-year survival, 0.79 at 3-year survival, 0.804 at 5-year survival. To assess whether the model was an independent indicator of EC, univariate and multivariate Cox regression analysis in the training set were executed, including clinical factors (age, stage, histological type and grade) and risk score. The Table 2 was exhibited after using multivariate Cox regression analysis. we got that high-risk group VS low-risk group: HR = 1.026, 95%*CI*1.015–1.037, *P* < 0.001, the results underscored this prognostic model possessed moderate independent prognostic value.

### 3.4 Validation of EC prognostic model in the testing set

To validate the predictive ability of this eleven-miRNA signature, the optimal cutoff value from the training cohort was also applied in the testing set. Samples in the testing cohort were divided into a high-risk group and a low-risk group likewise. It revealed that the survival time was decreased and the figure of dead people was up along with the elevated risk score by survival state plot (Figure 2d-2f). Therefore, the survival status was compliant with the previous inference. The K-M curve elicited the survival rate of the high-risk group was significantly inferior to that of the low-risk group. (*P* = 2.163e^−3^) (Figure 3b). The area under the ROC curve (AUC) was 0.721, 0.668, 0.713 at 1-year survival, 3-year survival, 5-year survival respectively (Figure 3d). Univariate and multivariate Cox regression analysis after adjusting for clinical characteristics were conducted to validate the independence of the model’s prediction in the testing cohort. The multivariate Cox regression analysis obtained from the Table 3 testified high-risk group VS low-risk group: HR = 1.001, 95%*CI*1.000–1.002, *P* = 0.019. To sum up, it was credible to proclaim the miRNA-based model harbored the independence to predict the prognosis of EC.

**Table 3.** The independent risk indicators of the EC using the univariate and multivariate cox regression analysis

Variables	Univariate analysis				Multivariate analysis		
	HR	95%CI	*p*		HR	95%CI	*p*
**TCGA training group**							
Age(≤60 vs >60)	1.338	0.717–2.497	0.360				
stage (stageI&stageII vs stageIII&stageIV)	5.283	2.918–9.566	<0.001		4.135	2.222–7.693	<0.001
Histological type(endometrioid vs Mix&serous)	2.551	1.411–4.611	0.002		1.794	0.924–3.483	0.084
grade(G3&G4 vs G1&G2)	3.436	1.599–7.386	0.002		1.949	0.828–4.590	0.127
riskScore(high vs low)	1.031	1.020–1.041	<0.001		1.026	1.015–1.037	<0.001
**TCGA testing group**							
Age(≤60 vs >60)	3.398	1.506–7.662	0.003		4.403	1.785–10.862	0.001
stage (stageI&stageII vs stageIII&stageIV)	3.081	1.676–5.662	<0.001		2.208	1.118–4.361	0.022
Histological type(endometrioid vs Mix&serous)	3.295	1.788–6.072	<0.001		1.100	0.524–2.309	0.800
grade(G3&G4 vs G1&G2)	3.875	1.716–8.749	0.001		3.006	1.173–7.705	0.022
riskScore(high vs low)	1.001	1.000–1.002	0.012		1.001	1.000–1.002	0.019

### 3.5 Survival analysis of subgroups between eleven-miRNA signature and clinicopathological features

**Figure 4.**
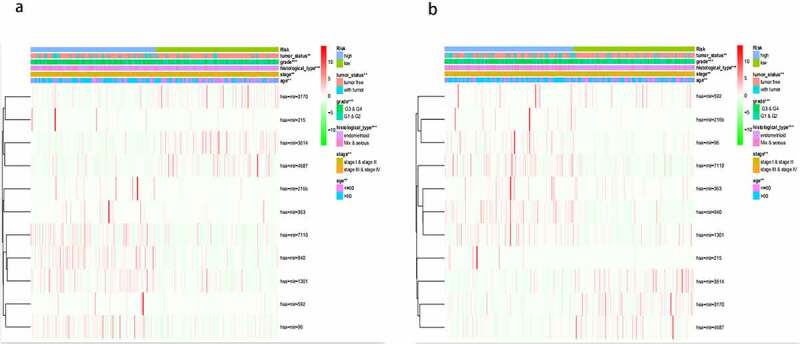
The difference of clinicopathological features and 11 miRNAs expression level of the high-risk group and low-risk group between training cohort (a) and testing cohort (b)

**Figure 5.**
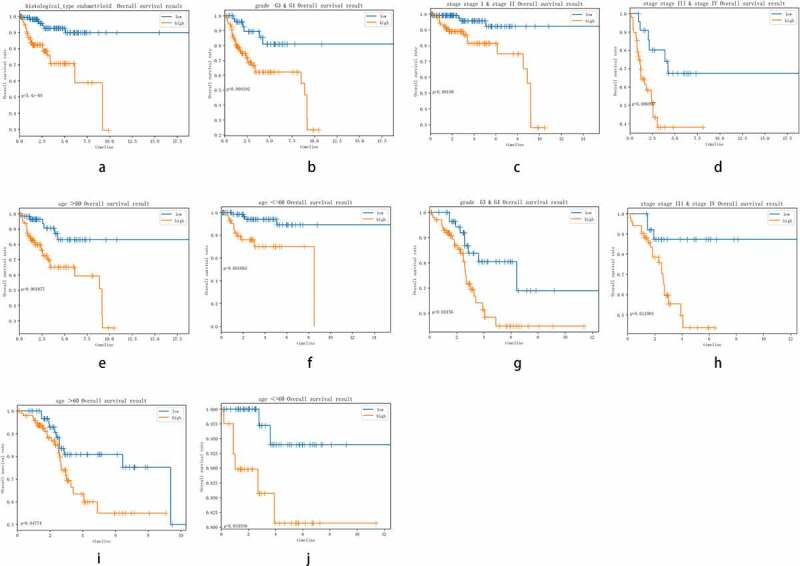
Comparison of the OS of EC among different clinical pathological features in high-risk group and low-risk group. A-F, the survival curves about tumor type, grade, stage, age between high-risk group and low-risk group were revealed in training cohort. G-J, the survival curves about age, grade, stage between high-risk group and low-risk group were verified in testing cohort

The clinicopathological features included grade (G3&G4, G1&G2), histological type (endometrioid, Mix&serous), stage (stageI&stageII, stageIII&stageIV) and age (≤60, >60). The distribution of these clinicopathological characteristics and the expression level of 11 miRNAs between the high-risk group and the low-risk group in the training cohort and testing cohort were presented in Figure 4a and 4b respectively. Subsequently, Kaplan–Meier curves were conducted for each subgroup, we could obtain the difference of OS for each clinicopathological variable through comparing risk stratification in the training set. Obviously, patients with low-risk had strikingly better OS than those with high-risk in endometrioid (*P* = 5.4e^−5^), grade G3 & G4 (*P* = 0.003192), stage I & stage II (*P* = 0.00198), stage III & stage IV (*P* = 0.006937), age>60 (*P* = 0.001877), age≤60 (*P* = 0.001002) (Figure 5a-5f). The robust predictive ability of this eleven-miRNA signature was also validated in the testing cohort. What’s more, the OS of patients with high-risk was still poorer than that with low-risk in age>60, age≤60, grade G3 & G4 and stageIII & stageIV, corresponding to *P* = 0.04774, 0.034936, 0.03156, 0.011903 respectively (Figure 5g-5j).

### 3.6 Comparison of the expression profile of DEMs in different clinicopathological signatures

To further investigate the gene expression profile, we applied t-test and *P* < 0.05 was the exclusion criterion. Consequently, the expression patterns of DEMs in each clinicopathological parameter were demonstrated in Table 4 and Table 5, corresponding to the training set and testing set. Similarly, the result of the training set was mainly in line with that of the testing set. Through in-depth thinking, among those clinicopathological signatures, histological type played a critical role in the expression pattern of DEMs.

**Table 4.** The *p*-value of DEMs in different clinical variables in the training set

Id	Age(≤60 vs >60)		Stage(III–IV vs I–II)		histological_type(endometrioid vs Mix&serous)		Grade (G3&G4 vs G1&G2)	
	t	p	t	p	t	p	t	p
hsa-mir-216b	−1.404	0.162	−1.767	0.082	0.277	0.782	1.079	0.282
hsa-mir-592	0.249	0.804	−0.58	0.564	1.466	0.144	−0.486	0.628
hsa-mir-3170	2.349	0.020	0.772	0.442	2.985	0.004	−1.946	0.053
hsa-mir-215	0.956	0.341	−0.798	0.428	−0.712	0.478	1.436	0.153
hsa-mir-940	−2.466	0.014	−1.361	0.176	−0.402	0.689	5.437	1.292e-07
hsa-mir-3614	0.69	0.491	2.06	0.042	4.676	6.762e-06	−3.929	1.241e-04
hsa-mir-1301	−1.337	0.182	−0.71	0.479	−2.773	0.007	1.807	0.072
hsa-mir-363	−1.066	0.288	−0.742	0.460	3.021	0.003	0.605	0.546
hsa-mir-4687	0.666	0.506	1.213	0.227	1.618	0.110	0.732	0.465
hsa-mir-96	1.72	0.087	−0.288	0.774	2.162	0.034	−1.336	0.183
hsa-mir-7110	−3.019	0.003	−0.949	0.345	−1.141	0.257	1.383	0.168
riskScore	−1.427	0.155	−1.925	0.059	0.884	0.378	2.237	0.027

**Table 5.** The *p*-value of DEMs in different clinical variables in the testing set

Id	Age(≤60 vs >60)		Stage(III–IV vs I–II)		Histological_type(endometrioid vs Mix&serous)		Grade (G3&G4 vs G1&G2)	
	t	p	t	p	t	p	t	p
hsa-mir-216b	0.066	0.948	−0.895	0.373	−1.987	0.051	2.261	0.025
hsa-mir-592	1.781	0.077	−0.088	0.930	3.779	1.996e-04	−1.789	0.075
hsa-mir-3170	2.027	0.045	0.484	0.629	3.8	1.959e-04	−1.261	0.209
hsa-mir-215	−1.01	0.341	−1.192	0.237	−1.146	0.256	0.892	0.374
hsa-mir-940	−2.045	0.042	−0.506	0.614	−0.964	0.337	3.465	6.304e-04
hsa-mir-3614	0.245	0.807	2.34	0.020	3.015	0.003	−3.203	0.002
hsa-mir-1301	−2.197	0.029	−0.973	0.332	−3.798	2.371e-04	1.524	0.129
hsa-mir-363	0.597	0.551	2.244	0.026	4.654	5.595e-06	−1.453	0.148
hsa-mir-4687	0.786	0.433	2.173	0.031	3.388	8.192e-04	0.171	0.864
hsa-mir-96	0.681	0.497	0.256	0.798	0.742	0.460	−1.235	0.219
hsa-mir-7110	−1.174	0.242	0.474	0.636	−1.525	0.130	2.029	0.044
riskScore	0.657	0.512	−1.277	0.206	−1.309	0.195	1.091	0.277

### 3.7 Assessment of the predictive value of the miRNA-based model with clinical parameters

**Figure 6.**
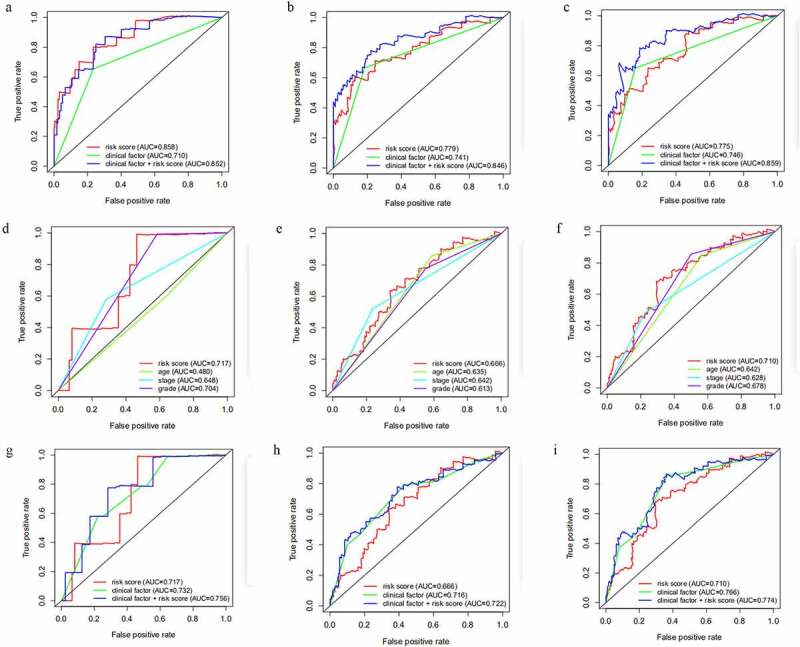
The AUC of risk factor and clinical characteristics to predict 1, 3, 5-year survival. A-C, The AUC of risk score, clinical factor and risk score+clinical factor was calculated via ROC curve in training cohort to predict 1, 3, 5-year survival. D-F, The AUC of risk score, age, stage and grade was acquired to assess the predictive value in 1, 3, 5-year survival in testing cohort. G-I, The AUC of risk score, clinical factor and risk score+clinical factor was compared to ensure the most optimal model to predict the prognosis in testing cohort

In order to acquire a better prediction capacity, we imagine combining the prognostic model with different clinicopathological features to investigate and compare the prediction ability. Then time-independent ROC curves were delineated to evaluate. The AUC of the risk score was better than that of clinical factor (stage) (risk score-AUC: 0.858, 0.779, 0.775 at 1, 3, 5 years; clinical factor-AUC: 0.710, 0.741, 0.746 at 1, 3, 5 years; risk score+clinical factor-AUC: 0.852, 0.846, 0.859 at 1, 3, 5 years (Figure 6a-6c). Meanwhile, it could infer model+clinical factor (stage) to predict 1-year survival was meaningless compared to single model, but more efficient in predicting 3-year survival and 5-year survival.

Subsequently, we assessed the robust predictive power in the testing set. The ROC curves (Figure 6d-6f) for risk score and clinicopathological traits shed light on the risk score (AUC: 0.717, 0.666, 0.710 at 1, 3, 5 years) had a higher prediction ability in comparison with age (AUC: 0.480, 0.635, 0.642 at 1, 3, 5 years), stage (AUC: 0.648, 0.642, 0.628 at 1, 3, 5 years) and grade (AUC: 0.704, 0.613, 0.678 at 1, 3, 5 years). Further study indicated the risk score+clinical factor-AUC was the most outstanding in predicting the survival, while the risk score-AUC was the minimum (risk score-AUC: 0.717, 0.666, 0.710 at 1, 3, 5 years; clinical factor-AUC: 0.732, 0.716, 0.766 at 1, 3, 5 years; risk score+clinical factor-AUC: 0.756, 0.722, 0.774 at 1, 3, 5 years (Figure 6g-6i). Taken together, the combination of the model and the clinical factor had more advantages in predicting the prognosis of endometrial cancer.

### 3.8 Functional enrichment analysis

**Figure 7.**
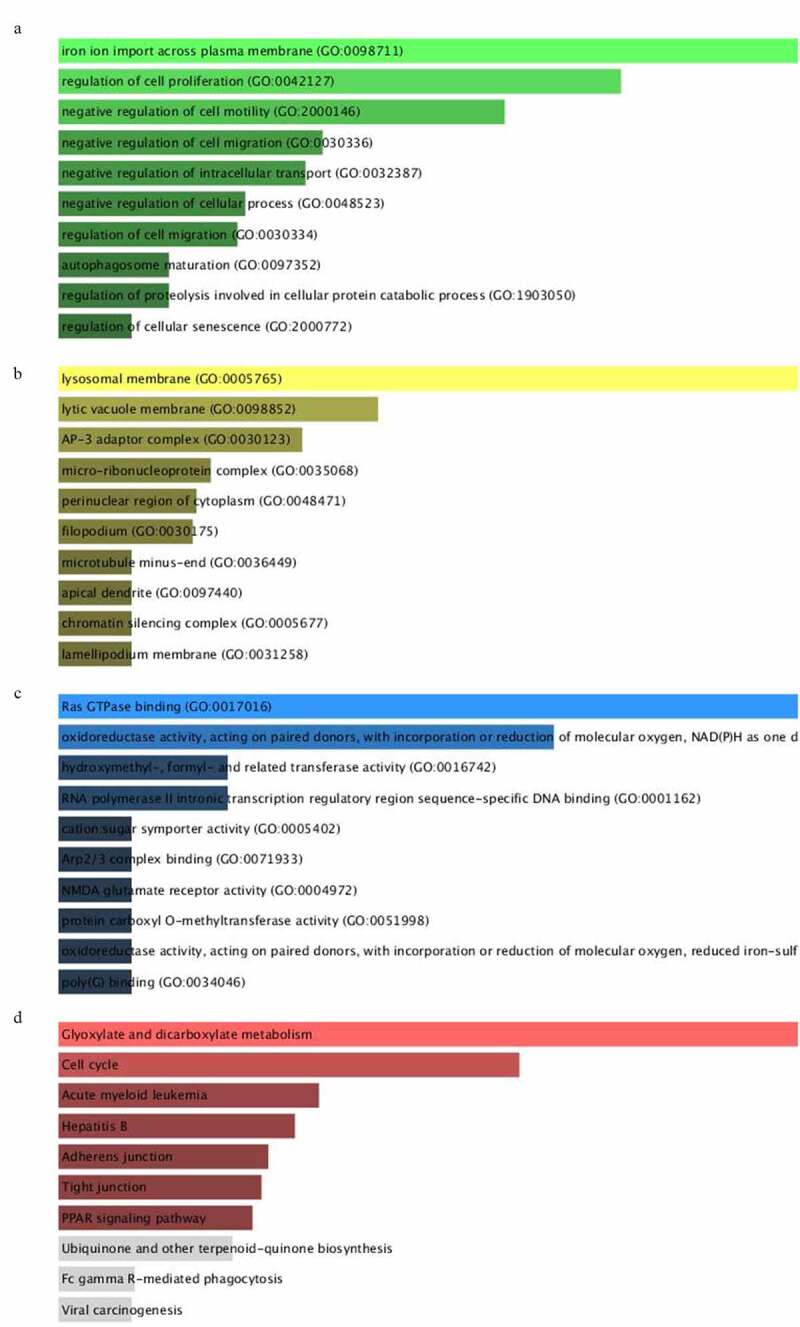
GO and KEGG functional enrichment analysis of targeted genes mediated by 11 microRNAs. A-C, denoted biological process (BP), cellular component (CC) and molecular function (MF). D, denoted KEGG pathway

To further research the molecular biological mechanisms of the eleven miRNAs, 94 target genes of them were emerged by using miRDB, miRTarBase and TargetScan. Subsequently, enrichr database was employed to unfold the GO biological function and KEGG pathway enrichment analysis. The GO analysis constituted of biological process (BP), cellular component (CC) and molecular function (MF) were shown in Figure 7, which exhibited the top 10 enriched items. The GO-BP analysis (Figure 7a) implied these target genes mainly enriched in iron ion import across plasma membrane (GO:0098711), regulation of cell proliferation (GO:0042127), negative regulation of cell motility (GO:2,000,146). The GO-CC analysis (Figure 7b) revealed these genes were significantly enriched in lysosomal membrane (GO:0005765), lytic vacuole membrane (GO:0098852), AP-3 adaptor complex (GO:0030123). For GO-MF analysis (Figure 7c), these target genes remarkably participated in Ras GTPase binding (GO:0017016), oxidoreductase activity (GO:0016742), RNA polymerase II intronic transcription regulatory region sequence-specific DNA binding (GO:0001162). On the other hand, the KEGG enrichment analysis indicated target genes were involved in the signaling pathway of glyoxylate and dicarboxylate metabolism, cell cycle, acute myeloid leukemia, hepatitis B and other pathways (Figure 7d).

### 3.9 The expression levels of prognosis-related miRNAs in paired tissues

**Figure 8.**
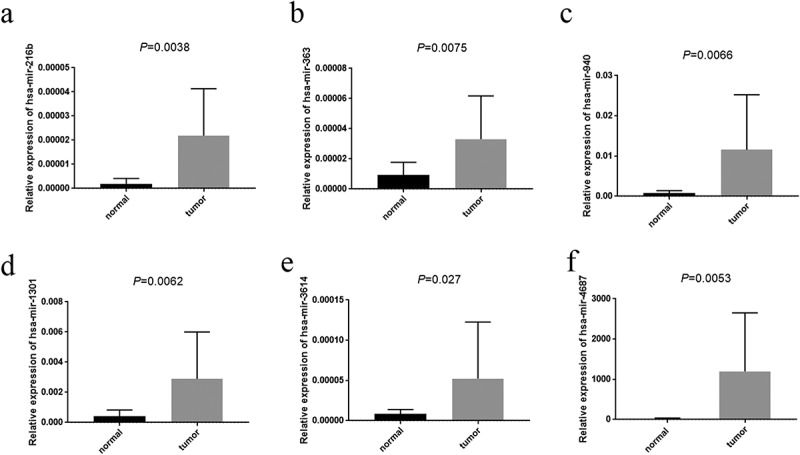
The expression of prognosis-related miRNAs. A-F represented hsa-mir-216b, hsa-mir-363, hsa-mir-940, hsa-mir-1301, hsa-mir-3614 (*P* = 0.027) and hsa-mir-4687 respectively. x-axis represented tissue type, y-axis represented the expression of each miRNAs comparative to U6

To verified the expression tendency of 11miRNAs in EC compared to normal tissues, we applied qRT-PCR to assess the expression level of them between 16 endometrial cancer tissues and paired 16 normal tissues. The results were exhibited in Figure 8. Analyzing with the coefficient of each miRNA, we acknowledged that the expressions of hsa-mir-216b (*P* = 0.0038), hsa-mir-363 (*P* = 0.0075), hsa-mir-940 (*P* = 0.0066) and hsa-mir-1301 (*P* = 0.0062) were prominently high-expressed in EC tissues, which was satisfied with our inference. However, the expressions of hsa-mir-3614 (*P* = 0.027) and hsa-mir-4687 (*P* = 0.0053) were also highly expressed in the tumor tissues compared with normal tissues which was contrary to our bioinformatics analysis.

## 4. DISCUSSION

Endometrial cancer is the most common gynecological tumor with a high degree of malignancy. On account of the limited therapy strategies for metastasis in patients with EC, there was a 15%-20% recurrence rate after surgery [24]. Therefore, establishing a prognostic signature with high specificity and sensitivity is desired and urgent, which could assist the selection of treatment strategy, promote the survival rate and contribute to better long-term prognosis.

Current studies manifested miRNAs functioned multiple roles in EC initiation and progression. Zhou et al. demonstrated plasma-derived exosomal miR-15a-5p was a effective biomarker in EC early diagnosis[33,781,255]. Chang et al. testified metapristone could inhibit EC cell proliferation via targeting miR-492/Klf5/Nrf1 axis[33,413,440]. About drug resistance, Megumi Yanokura et al. verified microRNA‑34b enhanced EC cell sensitivity to paclitaxel[33,300,049]. Recently, cutting-edge studies have certified miRNA was related to tumorigenesis and progression and could be considered as a potential biomarker in diagnosis, recurrence, treatment and prognosis of tumors. According to previous reports, miRNA was associated with diagnosis, prognosis, recurrence and therapy in breast cancer and papillary thyroid carcinoma [25–28], prognosis and therapy in ovarian cancer and colorectal cancer [29,30], diagnosis and progression in pancreatic cancer [31], prognosis in hepatocellular carcinoma and lung cancer [32,33]. As far as endometrial cancer, we listed several reports as following: Multivariate analysis demonstrated the decreased expression of miRNA-152 and miRNA-101 lead to poor prognosis in endometrial serous adenocarcinomas [32]. High expression of miRNA-34a was an indicator of positive prognosis in EC [34]. MiRNA-200 c maintained low expression levels in the advanced stage endometrioid EC that might generate inferior survival compared to the early stage [35]. These previous studies were incomplete lacking a comprehensive predictive model.

To further conduct a better model to improve the prognostic prediction ability of EC patients, we comprehensively analyzed and identified the novel biomarkers. First, 514 samples were finally selected from the TCGA database and were divided into a training set and a testing set. Secondly, by using the univariate, LASSO and multivariate Cox regression analysis, OS-related eleven miRNAs were ensured. Thirdly, The survival analysis in the training cohort based on the median risk score demonstrated the high-risk group was prominently poorer than that of the low-risk group (*P* = 2.367e^−5^). ROC curves were presented to evaluate the accuracy of the model’s prediction. After that, we verified the miRNA-based model was an independent indicator to predict the prognosis of EC patients through univariate and multivariate Cox regression analysis in two cohorts respectively. All the results were validated in the testing cohort. Next, we divided the clinicopathological features into different subgroups, for each subgroup in the training set and testing set, it revealed that the OS of the low-risk group was full of superiority compared with the high-risk group. Finally, we intriguingly attested the combination of the model and clinical factor had a more precise presentation in predicting the prognosis of endometrial cancer than the single model or clinical factor via ROC curves in the training set, the result was also confirmed in the testing set.

We expect to discover the therapy target so as to provide a novel strategy. The miRDB, miRTarBase, TargetScan and Enrichr database were executed to explore downstream genes and their potential molecular biological mechanism. The GO terms implied these target genes mainly enriched in regulation of cell proliferation, negative regulation of cell motility, lysosomal membrane, Ras GTPase binding, RNA polymerase II intronic transcription regulatory region sequence-specific DNA binding and so on. While the KEGG pathway enrichment analysis hinted the target genes were implicated in cell cycle, acute myeloid leukemia and so on.

Further, hsa-mir-216b (*P* = 0.0038), hsa-mir-363 (*P* = 0.0075), hsa-mir-940 (*P* = 0.0066) and hsa-mir-1301 (*P* = 0.0062), which we analyzed the coefficients of them were positive, were highly expressed in EC tissues compared with normal tissues by employing qRT-PCR while the expressions of hsa-mir-3614 (*P* = 0.027) and hsa-mir-4687 (*P* = 0.0053) were also highly expressed in the tumor tissues which were adverse contrast to our bioinformatics analysis.

Accumulating evidence testified miRNAs acted significant roles in cell fate determination, proliferation, and cell death [36]. The miRNAs that we identified in this model were testified in other researches mostly. The over-expressed miR-216b restrained the cell proliferation, migration and invasion in HCC through the HBx-miRNA-216b-IGF2BP2 signaling pathway [37], a similar phenomenon was observed in colorectal cancer via targeting SRPK1 [38]. Whereas downregulation of miRNA-215 significantly inhibited cell proliferation, migration and invasion via Yin-Yang 1 in colon cancer [39]. Over-expressed miRNA-940 promoted proliferation, migration, and invasion of bladder cancer cell and inhibited cell apoptosis by mediating INPP4A or GSK3β and activating the Wnt/β-catenin pathway [40]. IGF2BP3 served as an antagonism could inhibit miRNA-3614 maturation, mediately resulted in preventing breast cancer cell growth through downregulating TRIM25 [41]. Upregulated miRNA-363 increased glioma cell viability and proliferation by adjusting the expression level of GAP-43, AKT,cyclin-D1 and other factors [42]. The miRNA-96 served as a tumor suppressor gene in pancreatic cancer, decreased cell invasion, migration and growth through inhibiting KRAS [43]. To sum up, the regulatory function of miRNA-215, miRNA-940 and miRNA-363 in former pieces of research were parallel to the results what we drew, while the miRNA-216b, miRNA-592, miRNA-96, and miRNA-3614 in other tumors functioned the opposite effects in contrast to our data. This reminded us these miRNAs might function as a two-edged sword in different tumor tissues and involved in variant mechanisms. Therefore, it is meaningful to further investigate the tumorigenesis in EC. It is a pity that miRNA-1301, miRNA-7110, miRNA-3170 and miRNA-4687 were lacking in reports, but as a challenge, it indicated we could catch these underlying targets to conduct an in-depth study to uncover their potential functions and complex regulatory mechanisms. Moreover, all these previous studies indicated the miRNA-based biomarker was effective in tumor prognostic prediction and possibly offer novel diagnostic and therapeutic strategies.

Compared with former research, this study performed superiority as we integrated the clinicopathological features with predictive miRNAs to give rise to more accuracy in evaluating survival. What’s more, the prognostic model might also serve as an indicator of the selection of treatment strategies. However, there are several limitations. Firstly, our study lacks experiment verification, it is based on the TCGA database, so the molecular mechanism should be proved in the future. After that, the results of it need to be further validated before it is permitted to apply. Thirdly, explicit mechanisms should be elaborated in the further study.

### Conclusion

In conclusion, an eleven-miRNA-based model could be regarded as an independent indicator for the prognosis of EC patients and harbored the forceful predictive capacity or potential treatment value.

## Data Availability

All data are included in the article.

## Abbreviation list

EC: endometrial cancer; TCGA: the Cancer Genome Atlas; K-M: Kaplan-Meier;

ROC: receiver operation characteristic; AUC: area under the curve; qRT-PCR:

real-time quantitative RT-PCR; OS: overall survival; DFS: disease-free

survival; DEMs: differentially expressed miRNAs; GO: Gene Ontology; KEGG:

Kyoto Encyclopedia of Genes and Genomes; BP: biological process; CC: cellular

component; MF: molecular function.

## DECLARATION

**Ethics approval and consent to participate**

Ethical approval for the study was granted by the the Ethics Committee of the First Affiliated Hospital of Nanjing Medical University, and the current study was performed in conform to the Declaration of Helsinki.

## Highlights

1. 11 miRNAs-based signature could predict OS and PFS of EC patients accurately.

2. The combination of the 11 miRNA-based model and the clinical factor had more precise prediction in EC prognosis.

3. The biological functions of these miRNAs were closely related to metabolism, cell cycle, and cell proliferation.
